# Detection of Chromosomal Structural Alterations in Single Cells by SNP Arrays: A Systematic Survey of Amplification Bias and Optimized Workflow

**DOI:** 10.1371/journal.pone.0001306

**Published:** 2007-12-12

**Authors:** Kazuya Iwamoto, Miki Bundo, Junko Ueda, Yoko Nakano, Wataru Ukai, Eri Hashimoto, Toshikazu Saito, Tadafumi Kato

**Affiliations:** 1 Laboratory for Molecular Dynamics of Mental Disorders, RIKEN Brain Science Institute, Wako, Saitama, Japan; 2 Department of Neuropsychiatry, Sapporo Medical University, Chuo-Ku, Sapporo, Japan; Ecole Normale Supérieure de Lyon, France

## Abstract

**Background:**

In single-cell human genome analysis using whole-genome amplified product, a strong amplification bias involving allele dropout and preferential amplification hampers the quality of results. Using an oligonucleotide single nucleotide polymorphism (SNP) array, we systematically examined the nature of this amplification bias, including frequency, degree, and preference for genomic location, and we assessed the effects of this amplification bias on subsequent genotype and chromosomal copy number analyses.

**Methodology/Principal Findings:**

We found a large variability in amplification bias among the amplified products obtained by multiple displacement amplification (MDA), and this bias had a severe effect on the genotype and chromosomal copy number analyses. We established optimal experimental conditions for pre-screening for high-quality amplified products, processing array data, and analyzing chromosomal structural alterations. Using this optimized protocol, we successfully detected previously unidentified chromosomal structural alterations in single cells from a lymphoblastoid cell line. These alterations were subsequently confirmed by karyotype analysis. In addition, we successfully obtained reproducible chromosomal copy number profiles of single cells from the cell line with a complex karyotype, indicating the applicability and potential of our optimized workflow.

**Conclusions/Significance:**

Our results suggest that the quality of amplification products should be critically assessed before using them for genomic analyses. The method of MDA-based whole-genome amplification followed by SNP array analysis described here will be useful for exploring chromosomal alterations in single cells.

## Introduction

Single-cell human genome analysis is critically important for basic and medical genetics. Somatic genomic differences are found in both normal cellular differentiation, as seen in the immune system, and in the progression of diseases such as cancer. In addition, genomic differences within neurons of an individual, as seen in aneuploidy, have also been proposed to contribute to neuronal complexity [1]–[3].

Technical difficulties have hampered genomic studies using single cells because many analytic techniques require considerable amounts of genomic DNA (gDNA). A number of whole-genome amplification (WGA) methods [4], [5] and their application to single cells have been developed to overcome this obstacle. However, single-cell WGA (S-WGA) methods are notoriously susceptible to strong amplification bias [6] such as the failure of amplification of one of the two alleles (allele dropout, AD) and excess amplification of one allele or unequal amplification of the two alleles (preferential amplification, PA). Conventional PCR-based WGA methods such as degenerate oligonucleotide primed PCR (DOP-PCR) [7]–[10], primer extension preamplification (PEP) [11]–[14], and the linker-adaptor ligation-based method [15], [16] have been used to amplify DNA extracted from a small number of cells. However, these methods do not allow researchers to examine the amplification bias in a genome-wide manner. The genome regions to be amplified are known to be biased and limited in the conventional PCR-based methods. In addition, amplified products obtained by PCR-based WGA methods are generally too short in length to apply genome-wide single nucleotide polymorphism (SNP) genotyping technologies. In one PCR-based S-WGA method [17], [18], a less-biased genome amplification was proposed. However, it is still difficult to apply genome-wide SNP genotyping analysis to the product obtained by this method, due to the random fragmentation of gDNA and short product length.

Recently, multiple displacement amplification (MDA)-based WGA with a phi29 DNA polymerase, which yields less-biased and longer (>10 kb) amplified products [19], has been applied to a small number of cells. In the case of MDA from a small number of cells, consistent genotype and chromosomal copy number (CCN) profiles can be obtained from about 1,000 to 1,500 cells, and well-optimized experimental conditions have already been established [20]–[24]. Although MDA-based WGA has also been applied to single cells [6], [25]–[27], the knowledge of amplification biases, such as the frequency, degree, and preference for genomic location in the S-WGA products at the single-cell level has been very limited, and their effects on down-stream analyses have been poorly examined until now.

Here we performed a high-density oligonucleotide SNP array analysis of the MDA-based S-WGA products from a lymphoblastoid cell line (LCL) and a cell line having a complex karyotype. We examined the nature of the amplification bias using genome-wide genotype information, and assessed its effect on the CCN analysis. We also developed a SNP array data analysis approach to discriminate CCN alterations from amplification bias artifacts, which will be useful for exploring the chromosomal alterations in single cells.

## Results

### S-WGA from LCL and Taqman genotyping assay

**Figure 1:**
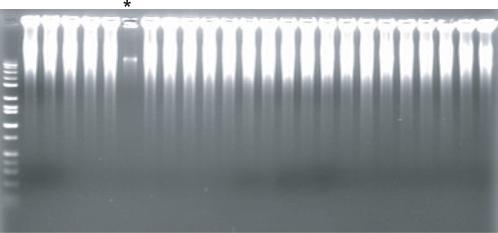
Visualization of the S-WGA products from a LCL by gel electrophoresis. We loaded 1 µl of each S-WGA reaction mixture obtained by protocol 2 (N = 24). A sample indicated by an asterisk was not selected for further analysis based on the results of the Taqman genotyping assay.

**Figure 2:**
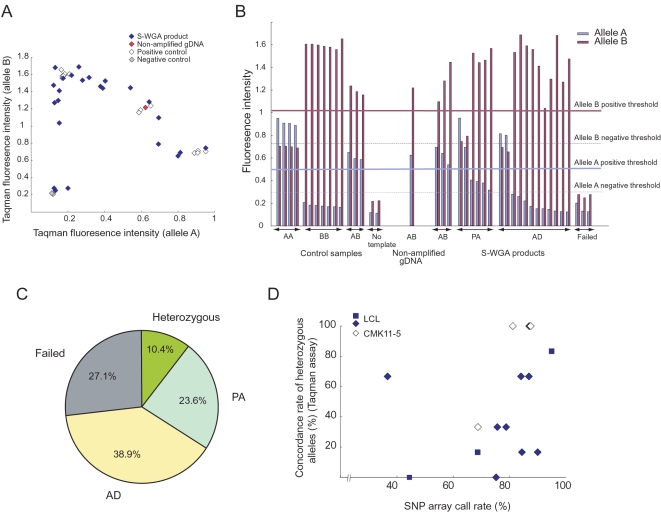
Amplification bias and manual genotyping of the heterozygous SNP. (*A*) Amplification bias revealed by the Taqman genotyping assay. The relative fluorescence intensities of alleles A and B in each sample with regard to rs1895694 are shown. Fluorescence intensities of the S-WGA products obtained by protocol 2 (N = 24) are shown. The results from another 5 heterozygous SNPs can be found in Figure S1. (*B*) Manual genotyping of rs1895694 in the S-WGA products. AB, heterozygous (allele AB); PA, preferential amplification; AD, allele dropout; Failed, failure in WGA. (*C*) Summary of the manual genotyping of the heterozygous SNPs. The percentage was calculated from a total of 240 data points obtained from genotyping of 6 heterozygous SNPs in 40 S-WGA products. (*D*) Concordance rate of the heterozygous SNPs by Taqman genotyping assay correlated with call rate on the SNP array. The data from 12 S-WGA products derived from a LCL and 3 S-WGA products derived from the CMK11-5 are shown. Genotyping results of 6 heterozygous SNPs (rs1895694, rs4706387, rs2074711, rs1007971, rs4140571, and rs2280964 for a LCL; rs1895694, rs7110302, rs11657541, rs1217617, rs9991, and rs2268248 for the CMK11-5) by Taqman assay were used for calculation of concordance rate. In genotyping S-WGA products, the heterozygous SNP as well as PA-classified SNPs were considered to be concordant with non-amplified gDNA. The blue squares and diamonds indicate the S-WGA products obtained by protocol 1 and 2, respectively.

We performed MDA-based S-WGA in 40 single cells obtained from one LCL (**Figure 1**). Among the 40 single cells, 16 were processed by protocol 1 and 24 were processed by protocol 2 (see Materials and Methods). We did not observe degradation of the starting gDNA (data not shown) or differences in size distribution among the S-WGA products, except for one sample with a lower yield than the others (**Figure 1**). The S-WGA products were genotyped for 23 SNPs, covering all autosomes and X chromosomes, using a fluorogenic 5′-nuclease (Taqman) assay (**Table S1**). The non-amplified gDNA of this LCL was found to be homozygous for 17 SNPs and heterozygous for 6 SNPs. With regard to the 17 homozygous SNPs in the non-amplified gDNA, none of the genotyped alleles in the S-WGA products showed inconsistent genotypes. On the other hand, we observed various degrees of amplification bias in the S-WGA products, involving the 6 heterozygous SNPs in the non-amplified gDNA (**Figure 2A** and **Figure S1**). We therefore manually determined the genotypes of the heterozygous alleles based on the fluorescence intensities of the Taqman assay, and classified them into the following categories: heterozygous (allele AB), PA, AD or failure (**Figure 2B**). We found that only 10.4% of the genotyped SNPs showed up as heterozygous, and the other SNPs showed a failure or amplification bias. (**Figure 2C**).

Among the 40 S-WGA products genotyped, we selected a total of 12 products for the SNP array analysis. We found that the rate of successfully genotyped alleles (call rate) on the array was modestly correlated with the concordance rate of heterozygous SNPs by the Taqman assay, if both PA and AB were considered to be successfully genotyped as heterozygous (R = 0.467, **Figure 2D**).

### Genotype analysis of S-WGA products from LCL using SNP arrays

**Figure 3:**
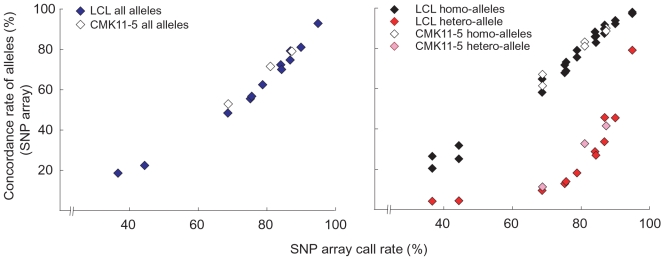
Concordance rate of genotypes correlated with call rate on the SNP array. Concordance rate of genotypes was determined by the global genotype data between non-amplified gDNA and S-WGA products. The results of all genotypes (*left*) and homozygous/heterozygous genotypes (*right*) are shown. Two data sets of S-WGA products from a LCL, with call rate<50%, were not used for further analysis.

We found that the call rate on the SNP array was strongly correlated with the global genotype concordance between S-WGA products and non-amplified gDNA obtained by SNP array (R = 0.982, **Figure 3**). The heterozygous SNPs in the non-amplified gDNA showed lower concordance (average±SD, 31.4±21.1%) among S-WGA products, compared with the homozygous SNPs (82.1±11.7%, **Figure 3**).

**Figure 4:**
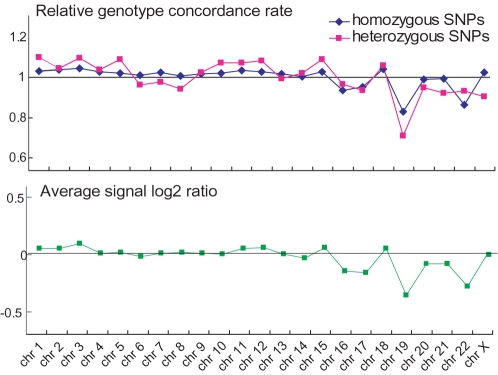
Genotype and CCN analyses in each chromosome. Y axes of the *top* and *bottom* panels indicate averaged relative genotype concordance (N = 10 array) and averaged signal log2 ratio (N = 10 array), respectively. Signal log2 ratio above and below 0 indicate gain and loss of CCN, respectively. Two apparently failed array data sets (call rate<50%) were excluded from the analysis.

To test whether the genomic location influences the amplification efficiency of MDA, we used the homozygous SNPs in the array data for analysis. The homozygous SNPs in the non-amplified gDNA were divided into three groups by genomic location: close to a centromere, close to a telomere (p arm or q arm), or located elsewhere. We found that SNPs close to centromeres or telomeres showed significantly lower genotype concordance between non-amplified gDNA and S-WGA products, compared with those located in other genomic regions (**Figure S2**). In addition, we observed that certain chromosomes, such as 19 and 22, tend to show discordant genotypes between non-amplified gDNA and S-WGA products (**Figure 4**).

We then examined whether genomic location influences the amplification bias using heterozygous SNPs in the non-amplified gDNA for analysis. In contrast to the homozygous SNPs, we observed that the heterozygous SNPs that showed discordant genotypes between non-amplified gDNA and S-WGA products were dispersed throughout the genome (**Figure S2**), suggesting a stochastic occurrence of AD in a genome-wide manner.

We then tested whether genome GC content (GC%) influences amplification bias. To this end, heterozygous SNPs in the non-amplified gDNA were used for analysis (**Figure S2**). We first excluded the heterozygous SNPs located close to the centromeres or telomeres described above. Among the remaining heterozygous SNPs, 2.6% (290 SNPs) were genotyped as homozygous or no calls in all of the 10 S-WGA products. We did not observe a preference for genomic location for these SNPs (data not shown), but found that the genome GC% slightly but significantly (P<0.01, t-test) affected the genotype concordance (**Figure S2**).

Among the 290 discordant SNPs, we used 154 whose genotype was homozygous in greater than five S-WGA products (i.e., non-no call SNPs) for further analysis. We then divided these SNPs into two groups: SNPs showing biased AD of one allele (i.e., the genotype call of each SNP tended to be exclusively one homozygous allele, at least 75% AA or BB) and other SNPs (i.e., the genotype call of each SNP was a mixture of two homozygous alleles, AA and BB). We found that a total of 74 SNPs showed a biased AD of one allele, and 80 SNPs showed non-biased AD. We did not observe the preference for genomic location for the SNPs showing the biased AD of one allele (data not shown). However, these SNPs showed statistically higher GC% (40.2%) compared with other SNPs (38.0%, P<0.05, t-test). These results suggest that genome GC% affects both the occurrence of AD and the preference of AD between two alleles to some extent.

### CCN analysis in the S-WGA products from LCL using SNP arrays

**Figure 5:**
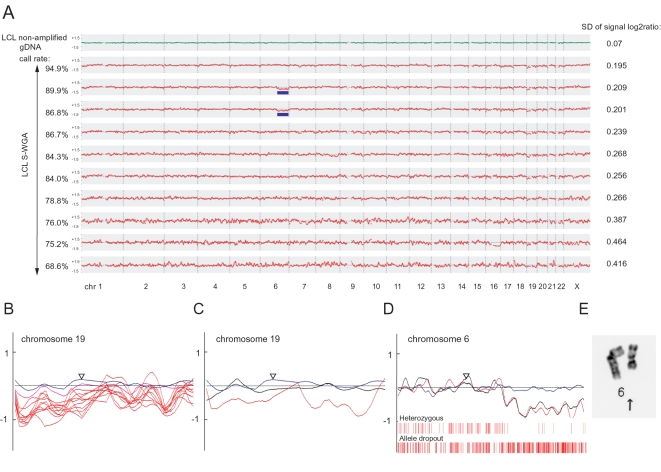
CCN analysis of the S-WGA products from the LCL samples. (*A*) CCN profiles of S-WGA products. Standard deviations of the signal log2 ratio are shown at the right. A genomic smoothing size of 2 Mb was used for analysis. Blue bars indicate the deletion in chromosome 6q. (*B*) CCN profiles of chromosome 19. Blue, pink and red lines indicate CCN data of non-amplified gDNA, WGA and S-WGA products, respectively. Profiles of 10 S-WGA products are shown. (*C*) Example of normalization of the CCN data of chromosome 19. The CCN data of chromosome 19 in an S-WGA product was normalized using the CCN data of the other nine S-WGA products as reference. Blue, red, and black lines indicate the CCN data of non-amplified gDNA, S-WGA product, and S-WGA product (normalized), respectively. (*D*) Example of the deletion of chromosome 6q in the S-WGA product. Weak signal log2 ratio of the 6q was still detected after normalization was applied (black line). Red bars indicate the location of the heterozygous SNPs in the non-amplified gDNA. Heterozygous and allele dropout indicate heterozygous and homozygous calls, respectively, on the array. (*E*) The result of karyotyping. Deletion of chromosome 6q was detected in 4 of 14 cells analyzed.

Accuracy of the CCN analysis, assessed by the standard deviation of the signal log2 ratio, was also strongly dependent on the call rate on the array (R = 0.885, **Figure 5A**). Consistency of the CCN data between S-WGA products and non-amplified gDNA was progressively lost in the S-WGA products showing lower call rates.

Similar to the genotype analysis described above, we also tested whether genomic location influences the CCN analysis by using both homozygous and heterozygous SNPs in the SNP array together. Genomic regions showing a frequent genotype discordance such as regions close to centromeres and telomeres showed statistically weaker signal log2 ratios compared with other genomic regions (**Figure S2**). We observed a strong correlation between genotype concordance and signal log2 ratio (R = 0.980 and 0.823 for homozygous and heterozygous SNPs, respectively). Specific chromosomes that showed discordant genotypes such as chromosomes 19 and 22 also showed weaker signal log2 ratios compared to other chromosomes (**Figure 4**).

To correct for these effects of amplification bias on CCN analysis, we developed a two-step examination of the candidate regions for chromosomal alterations. In the first step, CCN analysis of an S-WGA product was performed using a set of non-S-WGA products as reference, and candidate CCN alterations were identified. In the second step, CCN analysis of the S-WGA product was performed by using another set of S-WGA products as reference, and candidate regions were re-examined. By changing the reference, the CCN data can be successfully normalized in the insufficiently amplified regions, such as chromosome 19 and 22 (**Figure 5B, C**). Unexpectedly, we found that two S-WGA products showed a weak signal log2 ratio in a large part of chromosome 6q (20%), even after the normalization by other S-WGA SNP array data (**Figure 5D**). In that region, we also detected a considerable loss of heterozygous SNPs (**Figure 5D**). Subsequent karyotype analysis in this LCL revealed a deletion in chromosome 6q in four of the 14 cells analyzed (29%) (**Figure 5E**). This finding provided evidence that the weak signal log2 ratio observed in that region was not a technical artifact but reflects a true chromosomal deletion, demonstrating the appropriateness of our data analysis for the detection of chromosome structural alterations at the single cell level.

### CCN analysis in the S-WGA products from the CMK11-5 line using SNP arrays

The success in the detection of chromosomal deletion in the LCL allowed us to assess the ability of our approach to detect complex chromosomal abnormalities. We next performed CCN analysis of the S-WGA products from the CMK11-5 cell line, one of the derivatives of the CMK line. The CMK line was established from a patient with Down syndrome [28], and known to show complex hypo-tetraploidy. We confirmed that the CMK11-5 also showed complex hypo-tetraploidy and that this has a different karyotype compared with the parental CMK line (**Figure S3**).

With regard to the 23 SNPs used for genotyping by the Taqman assay, the non-amplified gDNA of CMK11-5 was found to be homozygous for 17 SNPs and heterozygous for 6 SNPs. After the S-WGA and subsequent Taqman assay of the 24 products, a total of 3 were selected for SNP array analysis. Similar to the LCL experiment, the call rates on the SNP array were strongly associated with the Taqman genotype concordance of heterozygous SNPs, when AB and PA-classified genotypes were considered to be concordant (**Figure 2D**).

**Figure 6:**
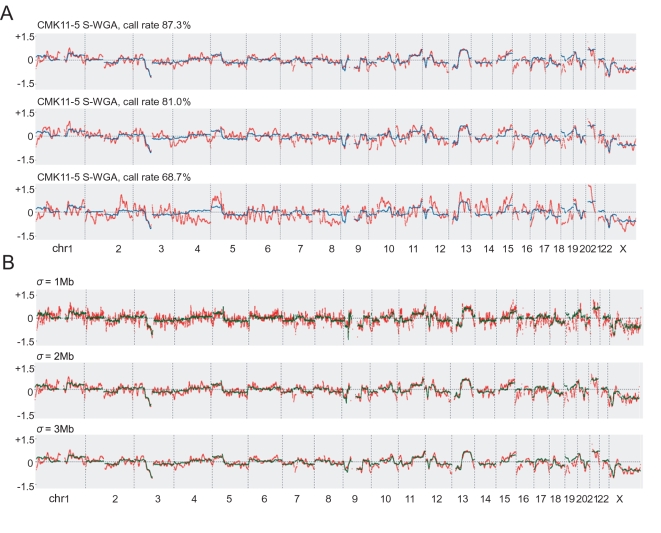
CCN analysis of the S-WGA products from the CMK11-5 cell line. (*A*) Results of the CCN analysis. A 3-Mb genomic smoothing size was used for analysis. Blue and red lines indicate CCN data of the non-amplified gDNA and S-WGA product, respectively. (*B*) Effect of the genomic smoothing size (σ) on the CCN analysis. Green and red lines indicate CCN data of the non-amplified gDNA and S-WGA product, respectively.

As predicted, concordance of the genotypes and the CCN profiles between the S-WGA products and non-amplified gDNA was dependent on the call rate on the array (**Figure 3** and **Figure 6A**). Nevertheless, the CCN profiles of 3 S-WGA products were very similar, supporting the reproducibility of the S-WGA and SNP array analysis in this cell line (**Figure 6A**).

We next analyzed various parameters in the CCN analysis using the Copy Number Analysis Tool (CNAT) 4.1 software. The optimal genomic smoothing size in the CCN analysis (σ, copy number state of each SNP was calculated using all flanking SNPs within 2σ to the left and right) is generally dependent on the type of analysis. We needed a genomic smoothing size of 2–3 Mb with about 80% of the call rate on the array to obtain consistent data with non-amplified gDNA (**Figure 6B**). For detecting chromosomal alterations in the CMK lines such as del(3)(p14), del(9)(p21) and additional chromosome 21, a genomic smoothing size of 1 Mb was sufficient (**Figure 6B**). Due to the limited resolution of the karyotype by the G-band and the existence of multiple marker chromosomes (**Figure S3**), determining the precise relationship between karyotype and CCN data was beyond the scope of the current study.

## Discussion

The large variability in amplification bias among S-WGA products, and the severe effect of amplification bias on the genotype and CCN analysis, suggest that the quality of S-WGA products should be critically assessed before starting down-stream analyses. It should be noted that size distribution and product yield did not differ among the S-WGA products, indicating that simple electrophoresis and DNA quantification cannot help the assessment of the product quality. Although only modest correlation was obtained, Taqman genotyping of heterozygous SNPs with consideration for PA was proven to be effective in predicting the SNP array call rate and screening for the products with potentially high quality (i.e., low rate of AD and less-biased genome amplification).

Because the amplification bias was seen, more or less, at every heterozygous SNP (**Figure S1**), we determined the genotypes arbitrarily by defining the thresholds in the Taqman genotyping assay (see Materials and Methods). In addition to AD, we determined PA in the genotyping. By considering the PA genotype as the heterozygous SNP, we observed correlation between the Taqman assay and SNP array call rate. We did not observe any correlation when we used other genotypes for calculation, such as homozygous SNPs, or heterozygous SNPs without consideration for PA (data not shown). Although changing the thresholds in the Taqman genotyping affected the results to some extent, the relationship between genotype concordance rate by the Taqman assays and the call rate on the SNP array (**Figure 2D**) was stably detected (data not shown). It should be noted that the Taqman genotyping assays were not suitable for quantitative purposes. However, considering that the fluorescence intensity was obtained at the endpoint of the assay, using positive controls of non-amplified gDNAs generally resulted in rigorous and reproducible fluorescence intensities for threshold determination.

Selecting the S-WGA products showing a concordance rate greater than 60% by Taqman assay of the heterozygous SNPs for the subsequent SNP array analysis generally resulted in call rates of at least 80% (**Figure 2D**). SNP array data with such a high call rate ensure a relatively low level of AD (average genotype concordance in the heterozygous SNPs was 41.7%, **Figure 3**) and a precise CCN profile for single cells with complex chromosomal structural alterations (**Figure 6**). It should be noted that we used 6 heterozygous SNPs for calculation. Among the 40 S-WGA products from the LCL, only about one-fourth of the products showed more than 60% concordance by the Taqman assay (data not shown). Increasing the number of heterozygous SNPs for the Taqman assay will improve this predictive ability. In addition, it has been recently reported that amplification bias in MDA can be ameliorated by reducing the reaction volume using nanoliter reactors [29]. Such approaches will enable researchers to more easily screen high quality S-WGA product.

We found that genomic location of the homozygous SNPs that showed discordant genotypes in the S-WGA products was mainly localized to the regions close to centromeres or telomeres (**Figure S2**). It has been known that repetitive genomic regions such as telomeres and centromeres tend to be underrepresented by MDA [19].

In addition, we observed that chromosomes such as 19 and 22 showed weak signal log2 ratio in the CCN analysis. Although the reason for this is not clear, this may not be specific to S-WGA, but rather a characteristic of the MDA-based WGA to some extent, because the signal log2 ratio in the WGA product using 10 ng of non-amplified gDNA as starting material also showed a slightly decreased signal log2 ratio in chromosome 19 (**Figure 5B**). It has been reported that specific chromosomal regions such as 1q42, 4q35, and 6p25 showed loss of representation after MDA [30], although these regions were not replicated in our previous study [31] or in the current study using S-WGA products. Recent array CGH analysis using the MDA products from a small number of cells isolated by laser capture-microdissected cells also revealed a reproducible biased-amplification of the genomic regions including telomeres and some chromosomes [23], [24]. In addition, in recent S-WGA studies, insufficient chromosome amplifications in the S-WGA products were found in both PCR- and MDA-based WGA using array CGH techniques [25], [32], [33], suggesting that some chromosomes are difficult to amplify by the S-WGA experiments.

Although we observed that genome GC content affects both occurrence of AD and preference of AD between two alleles to some extent, we found that AD occurred throughout the genome in addition to the insufficiently amplified genomic regions. These results indicate that genotyping the heterozygous SNPs at the single-cell level for medical purposes should be done cautiously, even in cases where high-quality S-WGA products can be used for analysis.

Unlike the array CGH analyses [18], [25], [33], SNP array analysis allowed us not only to carry out high-resolution CCN analysis, but also to apply critical quality assessment using genotype information. In addition, our two-step examination of the candidate regions for chromosomal alterations will effectively discriminate CCN alterations from biased amplification. A similar normalization principle utilizing the reproducible biased-amplification in MDA has also been reported in array CGH analyses [23], [33].

In summary, by using our optimized protocol in single cells from the lymphoblastoid cell line, we successfully detected a chromosomal deletion that was previously unidentified. In addition, we successfully obtained reproducible CCN profiles of single cells from the CMK11-5 cell line with a complex karyotype. The MDA-based S-WGA followed by the SNP array analysis described here will be useful for exploring chromosomal alterations in single cells.

## Materials and Methods

### Cell culture and karyotyping

A LCL, which was established by standard methods from a Japanese female subject, was maintained and cultured as described previously [34]. The CMK11-5 cell line was purchased from the Japanese Collection of Research Bioresources (JCRB) and cultured according to the provider's instructions. Before the S-WGA experiments, cell cultures were retrieved and washed once with phosphate-buffered saline (PBS). Karyotype of the LCL and CMK11-5 was determined by the G-band method (Japan SRL, Tokyo, Japan).

### Manipulation of single cells

Two areas for the single-cell experiments, each equipped with pipets, tubes and all reagents and instruments, were prepared in different rooms. To prevent contamination from external DNA to the reagent, one area was reserved for the preparation for reaction mixtures only, and nucleic acids were not handled there. Subsequent experiments were done in the other area, which was equipped with a UV PCR Workstation (UVP, Upland, CA, USA). Whenever possible, all equipment was UV irradiated for 30 min before the experiment began. Single cells were retrieved by mouth-controlled pipetting with a fine hand-drawn microcapillary tube under a stereoscopic microscope.

### S-WGA reaction

Two protocols (protocol 1 and 2) for S-WGA using a GenomiPhi V2 kit (GE Healthcare Life Sciences, Piscataway, NJ, USA) were provided by the manufacturer. They were similar to the method previously reported [6], [35]. Protocol 1: Single cells were transferred into a PCR tube containing 3 µl of sample buffer. The 1.5 µl of lysis solution (0.4 M KOH, 10 mM EDTA, and 100 mM DTT) was added to a tube and cells were lysed at room temperature for 10 min. 1.5 µl of neutralizing buffer (0.8 M Tris-HCl, pH8.0, and 0.4 M HCl), 1.5 µl of sample buffer, 7.5 µl of amplification mix (reaction buffer:enzyme mix = 9∶1) were added to a tube. The reaction mixture was then incubated at 30°C for 4 hours followed by heat inactivation at 65°C for 10 min. Protocol 2: Single cells were transferred into a PCR tube containing 3 µl of sample buffer. The 1.5 µl of lysis solution 2 (0.6 M KOH, 10 mM EDTA, and 100 mM DTT) was then added to a tube and cells were lysed at 30°C for 10 min. 1.5 µl of neutralizing buffer 2 (4∶1 mixture of 1 M Tris-HCl pH8.0 and 3 M HCl), 4.0 µl of sample buffer, and 10 µl of amplification mix (reaction buffer:enzyme mix = 9:1) were added to a tube. The reaction mixture was then incubated at 30°C for 4 hours followed by heat inactivation at 65°C for 10 min. In both protocols, the sample buffer, reaction buffer, and enzyme mix were included in the GenomiPhi V2 kit.

Despite extensive efforts, we sometimes observed amplified products from negative control samples (PBS, distilled water, or no addition of solution). Although they could not be distinguished from amplified products derived from single-cell samples by the electrophoresis or DNA quantification assays, a subsequent Taqman genotyping assay revealed no signals from them at all 23 SNPs (see below). Therefore, we concluded that amplification did not originate from human DNA, but originated from the primers included in the kit, or contamination of bacterial DNA included in the kit. Similar observations were also reported by others [6].

In obtaining the S-WGA products in the LCL, either protocol 1 or 2 was used. We did not find considerable differences between the two protocols with regard to the yield (protocol 1, 6.65±0.48 µg, N = 16; protocol 2, 6.63±0.52 µg, N = 24), Taqman genotyping assay and SNP array results (**Figure 2**). For obtaining the S-WGA products from the CMK11-5 line, we used protocol 2.

### SNP genotyping by Taqman assay

A total of 23 SNPs (one SNP for each autosome and chromosome X, see **Table S1**) were chosen by the following criteria. 1) SNPs were not included in the known copy number variations and 2) the minor allele frequency in the Japanese population was above 0.1. Genotyping was performed using Taqman assays (Applied Biosystems, Foster City, CA, USA) with an ABI PRISM 7900HT (Applied Biosystems). Probes and Universal PCR Master Mix were obtained from Applied Biosystems. In every genotyping assay, we included the S-WGA products as well as two negative controls (distilled water), 14 subjects for positive controls as described below, and a non-amplified gDNA sample. In genotyping the S-WGA products and positive control samples, 1 µl of the 5-times diluted S-WGA reaction mixture (about 70 ng) and 10 ng of gDNA, respectively, were used as template.

### Selection of the control samples for Taqman assay

To search for DNA samples suitable to serve as technical controls in the Taqman assay of S-WGA products, we genotyped a total of 40 Japanese lymphoblastoid DNA samples. Among the 40 DNA samples, 14 were selected for the quality control of the Taqman assay. These DNA samples were chosen so that all three genotypes (AA, AB, and BB) per SNP were covered by at least two subjects.

### Manual determination of the genotyping by Taqman assay

In manual determination of the genotypes of the S-WGA products, we arbitrarily defined the following four types of thresholds, based on the fluorescence intensities (FI) of the control samples. The positive allele A threshold was defined as follows: (average FI of allele A in the heterozygous alleles of the control samples)–3 * (standard deviation of FI of allele A in the heterozygous alleles of the control samples). Above this threshold, allele A in the S-WGA product was considered to be amplified. The negative allele A threshold was defined as follows: (average FI of allele A in the homozygous allele B of the controls)+5* (standard deviation of FI of allele A in the homozygous allele B of the controls). If the FI of allele A in the S-WGA product was below this threshold, allele A was considered to have dropped out of the amplification. The positive and negative allele B thresholds were determined the same way. Based on these thresholds, genotyping of the heterozygous SNPs in the S-WGA products were classified into 4 categories: AB (heterozygous, FI of both alleles were above the positive thresholds), PA (FI of one allele was above the positive threshold, while that of the other was not above the positive threshold but greater than the negative threshold), AD (FI of one allele was above the positive threshold, while that of the other was below the negative threshold), or failed (FI of both alleles were below the negative thresholds). An example of manual determination of genotyping is shown in **Figure 2B**. In genotyping the homozygous SNPs in the S-WGA products, we did not observe novel allele creations (for example, we never observed high FI for allele A in a homozygous allele B genotype).

### SNP array and genotype data analysis

Array data was deposited in the gene expression omnibus database (accession no. GSE8567). An Affymetrix 50KXba chip, which contains the probe sets for about 59,000 SNPs, was used for SNP typing. The SNP array data of the non-amplified gDNA of the LCL and the WGA product using 10 ng of LCL gDNA, were previously reported [31]. The S-WGA products of LCL and CMK11-5, as well as non-amplified gDNA of CMK11-5, were used for the SNP array analysis in this study. The experiment was performed according to the manufacturer's protocol. Regardless of the source of DNA, 250 ng of DNA was used as starting material. The raw SNP array data was processed by Affymetrix GeneChip Genotyping analysis (G-TYPE) software 4.1. The genotype was determined by the Dynamic model based algorithm in the G-TYPE software. Human genome reference of NCBI build 36 was used for analysis. Relative genotype concordance was calculated as follows: In each S-WGA array data set, genotype concordance rate was calculated by chromosome, and then concordance rates were divided by the average concordance rate (similar to per-chip normalization). Averaged relative genotype concordance is plotted in **Figure 4**.

### CCN analysis

CCN analysis was performed using the CNAT4.1 (Affymetrix). Typically, we performed unpaired sample analysis, in which one S-WGA SNP array dataset was used as the sample and eleven SNP array datasets from female subjects were used as reference. Reference samples were obtained in an Asian population used in the HapMap project. Median scaling implemented in the software was used for per-chip normalization.

## Supporting Information

Table S1 (0.03 MB XLS)

Figure S1 Amplification bias in heterozygous SNPs revealed by the Taqman genotyping assay. The relative fluorescence intensities of alleles A and B are shown.
(1.47 MB EPS)

Figure S2 (A) Genomic location of the SNPs showing inconsistent genotypes or no calls in the S-WGA products compared with non-amplified gDNA. Left, homozygous SNPs; right, heterozygous SNPs. Among the 12 profiled LCL SNP array datasets, the 2 with the low quality (call rate<50%) were removed from this analysis. Red and green bars on the chromosomes indicate the SNPs showing low (30% or less) and high (70% and above) consistency, respectively. Arrowheads indicate centromeres. Note that SNPs were not located in the p arms of several chromosomes in the Affymetrix 50K Xba SNP array. (B) Statistical analysis of the concordance and signal log2 ratio. Concordance rates of homozygous SNPs, or signal log2 ratio of the SNPs, in the telomeres or centromere were compared with those in the rest of the chromosome. Telomere SNPs included all SNPs within 10Mb of the end. Centromere SNPs included all flanking SNPs within the span 10Mb to the left and right of the centromere. P values by the Student's t-test are given. (C) Genome GC content and genotype concordance of heterozygous SNPs.
(2.98 MB EPS)

Figure S3 Karyotype of the CMK11-5. The CMK lines were established from a patient with Down syndrome, and showed hypo-tetraploidy. The CMK 11-5 was established from the CMK. The composite karyotype of 10 cells of the CMK11-5 was as follows: 83-90 <4n>, XYY, add(X)(p22.1)[10], add(1)(p36.3)[9], add(1)(q21)[10], -2[10], add(2)(p21)*2[10], -3[8], add(3)(q11)[2], del(3)(p14)*2[10], -4[9], +add(5)(q11.2)[9], add(5)(q13)[10], -6[8], der(6)add(6)(p23)del(6)(q?)[10], der(6)add(6)del(6)[3], -7[8], -8[10], -8[6], dup(8)(q11.2q21)[10], -9[10], -9[10], -9[7], der(9)del(9)(p21)add(9)(q34)[7], +10[2], del(10)(q22q24)[10], del(10)[7], add(11)(p15)[10], der(11;17)(q10;q10)[10], -12[9], add(12)(p11.2)[10], add(12)[8], add(12)(p13)[10], -13[10], -13[5], -14[10], -14[3], -15[10], -15[7], -16[4], -17[10], 18[9], add(18)(p11.2)[2], add(18)(q23)[10], add(18)(q23)[10], -19[10], -19[6], add(19)(p13)[10], der(20)t(1;20)(q2?5;q1?2)*2[10], +22[3], +10-14mar.
(4.68 MB EPS)
